# Exploring Molecular and Clinical Dimensions of Glaucoma as a Neurodegenerative Disease

**DOI:** 10.3390/ijms26189109

**Published:** 2025-09-18

**Authors:** Sandra Carolina Durán-Cristiano, Gloria L. Duque-Chica, Viviana Torres-Osorio, Juan David Ospina-Villa, Alba Martin-Gil, Geysson Javier Fernandez, Gonzalo Carracedo

**Affiliations:** 1 Grupo de Investigación en Ciencias Básicas, Facultad de Medicina, Universidad CES, Medellín 050010, Antioquia, Colombia; sdurancod@gmail.com; 2 Clinica Oftalmologica de Antioquia, CLOFAN, Medellín 050021, Antioquia, Colombia; 3 Facultad de Ciencias Exactas y Aplicadas, Institución Universitaria ITM, Medellín 050034, Antioquia, Colombia; gloriaduque@itm.edu.co; 4 Ingennova Research Group, Facultad de Ingenieria, Universidad CES, Medellín 050021, Antioquia, Colombia; vtorres@ces.edu.co; 5 Instituto Colombiano de Medicina Tropical, Universidad CES, Medellín 050021, Antioquia, Colombia; jospina@ces.edu.co; 6 Ocupharm Research Group, Universidad Complutense de Madrid, 28037 Madrid, Spain; amarting@ucm.es; 7 Grupo Biologia y Control de Enfermedades Infecciosas, Universidad de Antioquia, Medellin 050010, Antioquia, Colombia

**Keywords:** glaucoma, neurodegeneration, transcriptomic, cognitive function

## Abstract

Glaucoma is traditionally defined as an ocular disease characterized by progressive retinal ganglion cell degeneration, in some cases with elevated intraocular pressure (IOP), and optic nerve damage. However, growing evidence indicates that glaucoma shares critical features with neurodegenerative disorders, including Alzheimer’s and Parkinson’s diseases. This study aimed to explore the systemic nature of primary open-angle glaucoma (POAG) by integrating visual function, cognitive performance, and transcriptomic profiling. We conducted a multidimensional assessment of POAG patients and age-matched controls, accounting for demographic factors. Structural parameters included retinal nerve fiber layer (RNFL) thickness, measured using optical coherence tomography (OCT), and visual field indices mean deviation (MD) and pattern standard deviation (PSD). Cognitive function was evaluated across multiple domains, encompassing visual memory, executive function, processing speed, and verbal fluency. Additionally, transcriptomic analysis was performed from conjunctival samples to identify differentially expressed genes (DEGs) and enriched pathways. POAG patients exhibited significant RNFL thinning, which correlated with both visual field loss and cognitive impairments, particularly in terms of visual memory and executive function. Transcriptomic profiling revealed a distinct gene expression signature in POAG, including upregulation of *TTBK1* and *CCN2* (*CTGF*), genes associated with tau phosphorylation and extracellular matrix remodeling. Functional enrichment analysis indicated the involvement of neurodegenerative pathways, such as glutamate signaling, calcium signaling, and cell adhesion. Our findings support the reclassification of glaucoma as a neurodegenerative disease with both ocular and cognitive manifestations. Furthermore, biomarkers such as *TTBK1* and *CCN2* may serve as potential targets for early detection and neuroprotective therapy.

## 1. Introduction

Glaucoma is one of the leading causes of irreversible blindness worldwide, and is traditionally viewed as an ocular disease characterized by elevated intraocular pressure (IOP) and loss of retinal ganglion cells (RGCs). The prevalence of POAG ranges from 3.4% to 4% in Latin America [1] and was reported at 3.65% in Europe as of 2021 [2], indicating a substantial burden among the aging population. 

Nevertheless, in the last decade, a paradigm has redefined glaucoma as a complex neurodegenerative disorder that shares molecular and pathological features with central nervous system (CNS) diseases such as Alzheimer’s disease (AD) [3,4]. This reconceptualization has expanded the understanding of the condition’s scope beyond the eye, placing glaucoma within a broader neurological context.

Furthermore, a growing body of evidence suggests that glaucoma involves both functional and structural alterations, particularly in the visual cortex and other regions related to cognitive processing [5]. These findings emphasize that glaucoma is not merely an ocular disease; it may also impact cognitive function and overall neurological health, ultimately reducing quality of life. Several studies have reported cognitive impairment among glaucoma patients. For instance, in a meta-analysis, Wang et al. found that POAG patients are at higher risk of developing dementia and experiencing cognitive decline [6]. Also, research has shown lower cognitive performance in glaucoma patients, with cognitive screening scores being particularly reduced in those with normal-tension glaucoma (NTG) compared to high-tension glaucoma [7,8]. However, the relationship between glaucoma and cognitive impairment remains controversial.

Recent experimental studies have demonstrated the intricate molecular mechanisms underpinning glaucoma, revealing that RGCs’ degeneration, glial activation, and axonopathy are not an isolated ocular phenomenon, but are interconnected with systemic neurodegenerative pathways [9,10]. Particularly, oxidative stress, mitochondrial dysfunction, apoptosis, and neuroinflammation play relevant roles in disease progression [11].

The advent of transcriptomic technologies has revolutionized ocular research, enabling high-resolution profiling of gene expression changes in ocular tissues and fluids [12,13]. These approaches have not only deepened our understanding of glaucoma pathogenesis but have also uncovered potential biomarkers for early diagnosis and novel therapeutic interventions. For instance, in vitro transcriptomic analyses have identified gene signatures associated with oxidative stress and inflammation, offering promising targets for neuroprotective strategies.

Therefore, in this study, we aim to comprehensively characterize the molecular and clinical landscape of glaucoma by integrating transcriptomic profiling with cognitive and visual assessments in glaucoma patients. By bridging ocular and neurological perspectives, we seek to uncover novel biomarkers and therapeutic targets that may redefine the diagnosis and management of glaucoma as a true neurodegenerative disease.

## 2. Results

A total of 40 participants were included in the study: 20 diagnosed with POAG and 20 age-matched individuals without personal or familiar history of glaucoma (control group). The mean age in the POAG group was (59.76 ± 10.66) years, compared to (54.81 ± 6.05) years in the control group. There was no statistically significant difference in age between the two groups (*p* = 0.098). The sociodemographic characteristics of both groups are summarized in Table 1.

Interestingly, a proportion of participants in the POAG group had lower educational attainment than those in the control group. Specifically, 15% of the POAG group had a low level of education, 45% had a medium level, and 40% had a high level, compared to 40% with a medium level, and 60% with a high level of education in the control group. This difference was statistically significant (*p* < 0.001).

### 2.1. Visual Parameters

The average RGCs in POAG patients was mildly reduced compared to controls, but this difference lacked statistical significance (*p* = 0.192). The mean RNFL thickness for POAG patients was 95.46 ± 15.73 μm; similarly, RNFL Thickness Sup was thinner in POAG patients at 93.46 ± 21.99 (*p* ≤ 0.001). In the optic nerve analysis, POAG patients exhibited an increased vertical Cup-to-Disc Ratio (C/D) of 0.69 compared to 0.49, indicating cupping due to optic nerve damage. Additionally, the neural rim area in POAG patients was slightly reduced, while cup volume significantly increased (*p* ≤ 0.001), further suggesting optic nerve head damage (Table 2). Furthermore, we found a significant positive correlation between average RNFL and RNFL Thickness Sup, as well as between RNFL Thickness Sup and optic nerve head (ONH).

In functional analysis, visual field assessments revealed significant differences between POAG patients and healthy controls. The mean deviation (MD) was more negative (−5.20 dB vs. −2.04 dB), a statistically significant finding (*p* = 0.036) indicating a global reduction in visual sensitivity. The visual field index (VFI) was lower in POAG patients (87.23% compared to 98.01% in controls); this difference was statistically significant (*p* = 0.035), suggesting a possible trend toward overall visual function decline. In addition, the pattern standard deviation (PSD) was significantly higher in POAG patients (3.80 dB vs. 2.55 dB; *p* = 0.019), indicating localized visual defects consistent with glaucomatous damage. These findings demonstrate that POAG patients experience both global (MD) and localized (PSD) functional loss, in concordance with structural damage. 

Pearson correlational analysis was performed (Table 3) to examine the relationship between structural variables (OCT parameters) and functional variables (VF parameters). The analysis revealed several statistically correlations. Notably, a weak positive, yet non-significant correlation was observed between the RNFL thickness and MD (*r* = 0.360), suggesting a trend in which greater RNFL thickness may be associated with better overall visual performance. When comparing the POAG patients with the control group, a more negative MD was identified in the POAG group (*p* = 0.036), reflecting RNFL thickness and a reduced neuroretinal rim. A significant negative correlation was found between RNFL thickness and PSD (*r* = −0.53, *p* = 0.032), indicating that greater RNFL thickness is related to less focal visual field damage. A similar, though not statistically significant, negative correlation was observed between superior RNFL thickness and PSD (*r* = −0.46, *p* = 0.069). This finding suggests that greater thickness in the superior area is associated with less focal disturbance in the visual field, which is consistent with the understanding that structural damage in glaucoma may manifest as an increased PSD.

### 2.2. Neuropsychological Findings

Significant differences in neurocognitive performance were observed between the POAG and control groups across multiple cognitive domains. As detailed in Table 4, patients with POAG demonstrated lower performance in visuospatial construction (*p* = 0.015) and visual memory (*p* = 0.001), as well as in processing speed and cognitive flexibility (*p* = 0.005 and *p* = 0.002 for Trail Making Test (TMT) A and B, respectively). A significant difference was also found in the TMT B–A score (*p* = 0.048). For the TMT analyses, four POAG patients who exceeded the maximum allowable time for valid test completion were excluded, in accordance with standard scoring guidelines.

#### 2.2.1. Cognitive Impairments

A detailed analysis revealed the following cognitive impairments in the POAG group:

- Attention and Executive Function: A substantial proportion (65%) of the POAG group exhibited prolonged completion times on TMT-A, with 50% showing markedly extended times on TMT-B. This indicates significant difficulties in attentional control and cognitive flexibility.
- Visual Memory: The majority of patients (85%) scored below the expected range on the Rey–Osterrieth Complex Figure (ROCF) delayed recall, reflecting severe nonverbal memory deficits.
- Visuoconstructive Planning: Additionally, 40% of patients performed below average on the ROCF copy task.
- Executive-Linguistic Functioning: A total of 55% of participants scored below normative expectations on phonemic and semantic fluency tasks.

#### 2.2.2. Correlation Analysis Between Visual and Cognitive Functions

As summarized in Table 5, moderate correlations were found between neurocognitive performance and both functional (VF) and structural (OCT) visual parameters. Perceptual-motor skills, assessed by the ROCF copy, were positively associated with global visual sensitivity (MD, *r* = 0.534, and *p* = 0.001).

Measures of executive functioning showed significant inverse correlations with RNFL superior thickness. Specifically, TMT B performance was negatively correlated with RNFL superior thickness (*r* = −0.480, *p* = 0.004), suggesting that reduced retinal nerve fiber layer integrity is linked to diminished executive functioning in POAG patients

ROCF memory was significantly associated with MD (*r* = 0.529, *p* = 0.001), PSD (*r* = −0.591, *p* = 0.000), and with multiple verbal fluency tasks (*p* < 0.01). Additionally, language-related fluency tasks were significantly associated with GCC thickness and RNFL metrics (*p* < 0.01), highlighting the contribution of retinal structural changes to higher-order cognitive performance.

Furthermore, significant correlations were observed among neuropsychological measures themselves, particularly between visuospatial memory (ROCF), executive functioning (TMT), and verbal fluency, suggesting an internally consistent pattern of cognitive impairment in POAG. These findings support the hypothesis that structural alterations in the retina, particularly in the RNFL and GCC, may reflect specific domain-related cognitive deficits in individuals with POAG.

### 2.3. Transcriptome Analysis Revealed and Data Analysis Differentially Expressed Genes in Glaucoma

**Figure 1:**
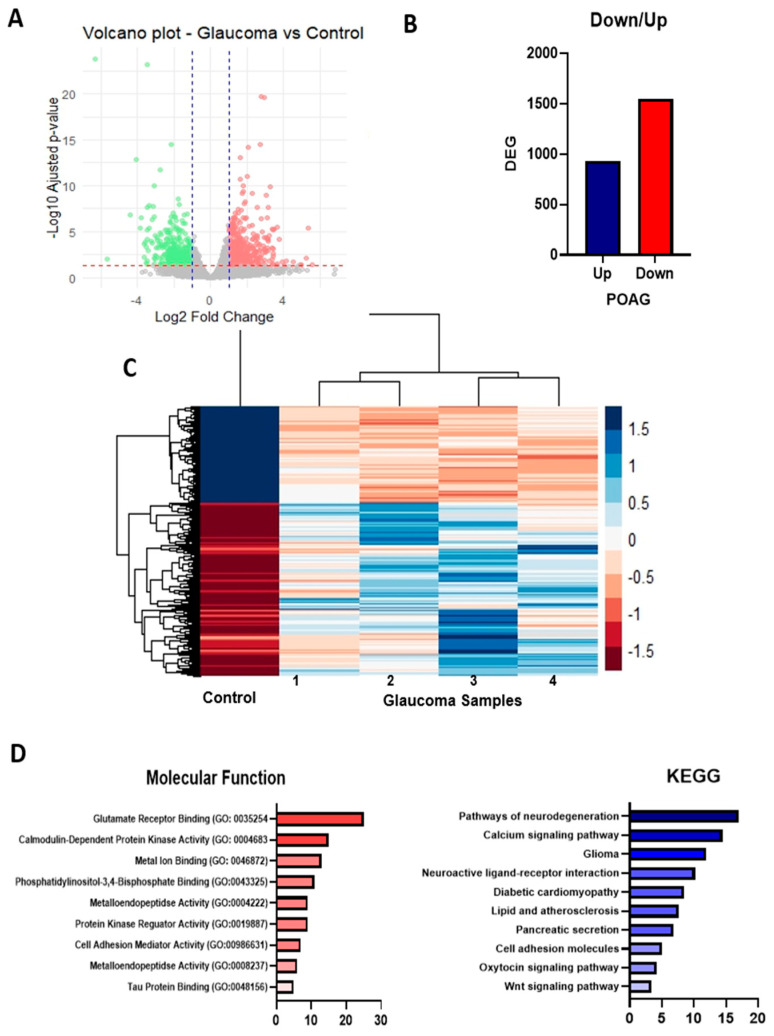
Differential gene expression analysis in POAG patient’s vs. control sample. The figure presents the identification of DEGs, (**A**) Volcano plot of DEGs in glaucoma patients and control. (**B**) Barr plot shows the total number of upregulated (blue) and downregulated (red) genes in glaucoma compared to control. (**C**) Hierarchical Heatmap. (**D**) Functional enrichment analysis of DEGs. Left panel (GO Molecular function) and right panel (KEEG pathway).

Previous transcriptomic studies in glaucoma have examined various models and tissue types. In the present study, RNA-seq analysis of epithelial conjunctival cells identified 29,210 genes, among which 2477 were differentially expressed genes (DEGs). Of these, 1548 genes (62%) exhibited significant upregulation, while 929 genes (38%) showed significant downregulation in POAG patients compared to healthy controls (logFC > 0.5; adjusted *p* < 0.01) (Figure 1B). Some genes appeared at the extremes of the graph (Figure 1A), suggesting relevant changes in expression associated with the glaucomatous condition.

According to the DEGs, a hierarchical heatmap indicated that the gene expression profile of the control sample differs markedly from that of the POAG samples, suggesting a systematic change in gene expression related to glaucoma. In the dendrogram, the POAG samples cluster together, reflecting a shared transcriptomic pattern associated with this condition. Upregulated genes (dark blue) are shown in (red/pink) in POAG samples, while other genes exhibit an opposing profile, indicating significant alterations in the transcriptome linked to glaucoma. A consistent expression profile was observed within each group, further highlighting a clear separation between the POAG and control groups, which signifies distinct global gene expression profiles. These distinctions were visualized in a heatmap, illustrating consistent expression differences across individual samples (Figure 1C).

To gain deeper insights into the functional implications of these DEGs, a GO-enriched analysis was conducted. This analysis revealed several overrepresented molecular functions, with key GO terms among the upregulated genes including glutamate receptor binding, metal ion binding, cell adhesion mediator activity, tau protein binding, and calmodulin-dependent protein kinase activity (Figure 1D).

Additionally, KEGG pathway analysis identified significantly enriched biological pathways associated with neurodegeneration, calcium signaling, glioma, neuroactive ligand-receptor interaction, and Wnt signaling pathways (Figure 1D). These findings suggest that POAG may involve molecular alterations in neuronal signaling, synaptic regulation, and neurodegenerative processes, reinforcing the classification of glaucoma as a neurodegenerative disease. Other DEGs identified as upregulated include those involved in neuronal signaling (*CAMK2*, *TTBK1*, *CACNA1*, and *NBDF4*), cell signaling and growth factors (*CCN1*, *CNN2*, *GDF-1*, *KDR*, and *APCDD1*), inflammation and oxidative stress (*NOS2*, *NOX1*, *CDKN2*, and *TGFA*), and DNA/RNA modification (*APOBEC3* and *TRF5*). In contrast, downregulated genes are associated with cell proliferation and the cell cycle (*MKI67*, *ANLN*, and *FHL*), synaptic function (*GRIA4* and *CHRM3*), growth factors and hormone regulation (*BDNF*, *FGF-1*, and *IGFBP6*), as well as with cellular adhesion and the extracellular matrix (*FAT2* and *LAMB1*).

### 2.4. RT-qPCR Validates the Results of RNA-seq

**Figure 2:**
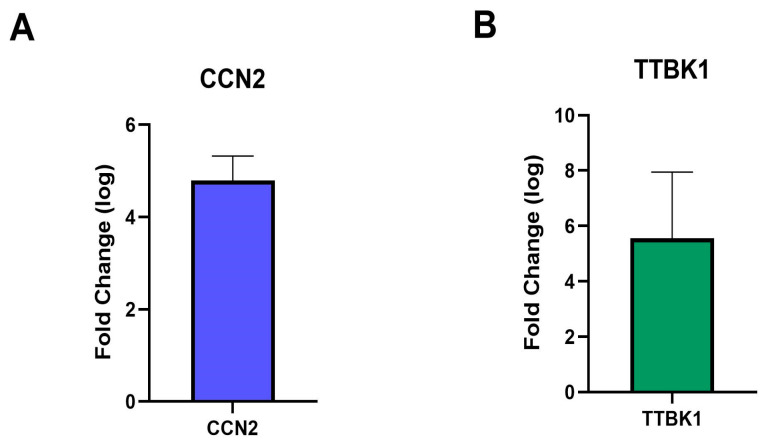
Log Fold Change of genes expression in POAG samples. Changes in the expression of genes *CCN2* (**A**) and *TTBK1* (**B**) in conjunctival samples from POAG patients. The mRNA levels measured by quantitative polymerase chain reaction (qPCR) are presented as the relative fold change normalized against the housekeeping 18S ribosomal gene. Both independent experiments showed the same trend in the expression of functional molecules by quantitative real-time PCR (qRT-PCR). Data are expressed as mean ± SD (*n* = 2).

Validation of RNA sequencing data was performed using the two upregulated genes showing in conjunctival epithelial cells from POAG patients. The findings demonstrated that the RNA levels of these genes, obtained through RT-qPCR, aligned with the RNA-seq results (Figure 1A). The expression changes in the two upregulated genes, including *CCN2*/*CTGF* and *TTBK1*, are summarized in Table 6 and illustrated in Figure 2.

All glaucomatous samples exhibited elevated expression of *CCN2* compared to the control, with fold changes ranging from 3.08 to 9.16, indicating moderate to strong upregulation of the gene. Similarly, *CCN2*/*CTGF* expression consistently increased across all glaucomatous samples, suggesting a steady upregulation that may reflect chronic activation of pro-fibrotic and inflammatory signaling pathways. In addition, *TTBK1* displayed a more variable expression profile; specifically, three samples exhibited strong upregulation.

Notably, tau protein binding (*TTBK1*) was also detected, indicating the selective non-covalent interaction with tau protein, a molecule implicated in AD physiopathology. This finding suggests a potential molecular connection between glaucoma and other neurodegenerative disorders, such as AD. In this context, KEEG pathway enrichment analysis showed that the most significantly represented pathway was related to neurodegeneration, alongside other relevant pathways linking Wnt signaling, cell adhesion, and glioma-associated signaling. These results further support the hypothesis that glaucoma, beyond being an ocular disease, may share common mechanisms with systemic neurodegenerative processes. In summary, these findings confirm the upregulation of *CCN2*/*CTGF* in glaucomatous samples, reinforcing its roles in tissue fibrosis and structural alterations, while the upregulation of *TTBK1* may be linked to neurodegenerative processes.

## 3. Discussion

Glaucoma has traditionally been regarded as an ocular condition characterized by the progressive degeneration of retinal ganglion cells and elevated IOP, which consequently damages the optic nerve [14]. However, growing evidence suggests it also exhibits characteristics of a neurodegenerative disorder, sharing pathobiological features with conditions such as Alzheimer’s and Parkinson’s diseases [15]. This study explores this broader perspective by integrating visual outcomes, cognitive assessments, and transcriptomic analysis, thereby offering a more comprehensive understanding of the systemic impact of glaucoma.

As expected, patients with glaucoma demonstrated significant RNFL thinning, indicating axonal degeneration of retinal ganglion cells. These changes correlate with visual field loss, a hallmark indicator of glaucomatous progression, reflecting a direct relationship between greater RNFL thinning and more negative MD values. These findings support previous research underscoring the clinical significance of diagnosing and monitoring glaucoma, particularly concerning its association with vision loss tied to RNFL thinning [16,17]. Furthermore, we identified a correlation between average RNFL and MD in POAG samples, emphasizing the importance of both structural and functional changes in the disease, despite no increased significance related to IOP.

The observed correlations between structural and functional parameters were moderate to weak, highlighting the inherent complexity of glaucoma. This disease features a heterogeneous progression where structural changes, such as RNFL thinning, and functional deficits in the visual field may not occur simultaneously or exhibit the same severity pattern [18]. The discrepancy in timing and intensity between structural and functional findings highlights the necessity for a comprehensive diagnostic and monitoring strategy that integrates both imaging modalities and functional and additional assessments to evaluate glaucomatous progression more accurately.

While mathematical models have been proposed to enhance the prognostic assessment of glaucoma, variability persists among patients [19,20]. Some individuals exhibit a distinct phenotype characterized by significant structural damage without corresponding visual functional loss, a phenotype that complicates early diagnosis and management [21]. This structure and function dissociation has prompted research into alternative biomarkers. Emerging evidence indicates that glaucoma is associated with changes in the CNS.

Studies utilizing animal models and cell cultures have explored how retinal damage may extend to cortical areas through disrupted retinal–cerebral connectivity [22,23,24]. In this context, Vidal-Villegas B et al. identified several biological events in glaucoma, including glutamate excitotoxicity, a mechanism also implicated in AD, with NMDA-induced retinal excitotoxicity leading to RNFL loss [25]. Similarly, Salobrar-Garcia et al. demonstrated significant RNFL thinning in an AD murine model [26], further supporting a shared degenerative pathway between glaucoma and central neurodegenerative conditions.

Neuroimaging studies have uncovered cortical alterations in glaucoma. Yu L et al. observed significant bilateral thinning of the anterior visual cortex, particularly in the calcarine sulci of POAG patients; these changes correlated with reduced RNFL thickness [27]. Similar structural deficits have been reported in the lateral geniculate nucleus (LGN) and lateral occipital gyrus, indicating widespread CNS involvement in glaucoma.

Importantly, RNFL thinning, a hallmark of ocular degeneration, is increasingly recognized not only as an ophthalmological biomarker but also as a potential indicator of broader CNS changes [28,29]. This association suggests a shared neurodegenerative pathway between the eye and the brain, highlighting the systemic nature of POAG. In this study, POAG patients demonstrated significantly lower neurocognitive performance compared to controls across multiple cognitive domains, including visuospatial construction, visual memory, executive functioning, and verbal fluency. Visual memory was most impaired, with 85% of patients scoring below the clinical thresholds on the ROCF recall test, indicating substantial difficulty in the encoding and retrieval of complex visual information.

Executive dysfunction was also evident in our cohort. Fifty percent of the POAG group showed impairments on the TMT-B, while 35–55% experienced deficits in verbal fluency, reflecting diminished cognitive flexibility and lexical access. Additionally, 65% of patients exhibited slowed processing speed on the TMT-A, indicating attentional inefficiency and reduced cognitive tempo. These findings corroborate prior studies that reported executive dysfunction in glaucoma patients using various assessment tools, including the Verbal Fluency Subtest of the D-KEFS [30], the Clock Drawing Test [31,32], and executive tasks like the Wisconsin Card Sorting Test (WCST) and Go/No-Go paradigms [33,34]. The convergence of evidence across methodologies underscores the presence of a specific executive-attentional deficit profile in glaucoma.

Moreover, we observed moderate correlations between cognitive performance and retinal measures, encompassing both structural (RNFL and RGCs thickness) and functional (MD and PSD) visual parameters. These associations suggest that retinal damage may serve as a proxy for cortical dysfunction, particularly in the frontal and temporal networks involved in executive, memory, and language functions. This finding supports the broader hypothesis that the retina acts as an extension of the brain, reflecting central neurodegenerative processes [35].

Our results are also in line with recent work by Garg et al. (2025), who reported significant RNFL thinning and poorer cognitive performance in POAG patients on global assessments (ACE-III, PGIMS) [33], along with greater difficulty in executive and memory tasks such as the WCST, Go/No-Go, and TMT.

Conversely, transcriptomic analysis revealed a distinct gene expression profile in POAG patients compared to the control group. Several differentially expressed genes were identified, many of which are linked to both glaucoma pathology and neurodegenerative processes. Notably, *TTBK1* (Tau Tubulin Kinase 1), which regulates tau phosphorylation, a key pathological hallmark in AD [36], was upregulated in glaucomatous samples. This suggests a potential mechanistic overlap, whereby aberrant tau signaling may contribute to both retinal and cognitive decline. These findings align with recent research in normal tension glaucoma (NTG), where an analysis of small extracellular vesicles (sEVs) from plasma indicated that mitochondrial dysfunction may trigger programmed axon death and activate neurodegenerative pathways [37]. Other neurotrophic factors, such as *BDNF* and *CTGF*, have also been implicated in both ocular and neurodegenerative disorders, further underscoring the biological connection between the brain and the eye. Indeed, new strategies have explored neuroprotective therapies to mitigate RNLF loss.

Despite some variability across POAG samples, dendrogram analysis revealed clear clustering of four POAG samples, indicating consistent expression profiles. Highly expressed genes in control samples showed a marked decrease in the POAG samples, while other genes exhibited the opposite pattern. This contrast underscores potential differential biomarkers that may have diagnostic or therapeutic relevance in glaucoma.

Indeed, structural deficits in the RNFL have been observed across multiple neurodegenerative diseases, including AD and Parkinson’s, mirroring the retinal changes seen in glaucoma [38,39]. Several neurodegenerative disorders have demonstrated structural deficits in the RNFL, echoing similar findings in glaucoma. In contrast, several studies explore neuroprotective strategies in glaucoma emphasize that protecting the inner retina is crucial for preventing damage to the outer retina [40], which has significant implications.

Among the most relevant genes, *CTFG* (also known as *CCN2*) was significantly upregulated in glaucomatous eyes. CTGF interacts with TGF-β and bone morphogenetic proteins (BMPs), promoting a shift towards increased TGF-β signaling and suppressed BMP activity [41]. This imbalance contributes to pathological changes in the trabecular meshwork (TM) cytoskeleton and ECM remodeling. In animal models, CTGF overexpression leads to elevated IOP and subsequent optic nerve axon loss [42]. Our study indicates increased RNFL thinning in POAG patients, which may correlate with axonal damage in the optic nerve, leading to functional vision loss and RNFL degeneration. In this context, trophic neuronal factors emerge as key elements in glaucoma pathophysiology, emphasizing the disease’s neural impact.

Furthermore, CCN2/CTGF is associated with fibrosis, extracellular matrix remodeling, and glial activation; processes that are prevalent in both glaucoma and neurodegeneration [41,43]. Functional enrichment analysis further indicated that differentially expressed genes were associated with glutamate receptor activity, metalloproteinase regulation, and cell adhesion, all of which are essential to maintaining synaptic integrity and modulating neuroinflammation.

Single-nucleotide polymorphisms (SNPs) in the *TTBK1* gene have been linked to AD, as this gene regulates and promotes tau phosphorylation and accumulation. Variants of *TTBK1* are implicated in tau aggregation and axonal degeneration [44]. Studies using murine models demonstrate that *TTBK1* overexpression and increased tau phosphorylation contribute to cognitive impairment [45]. Additionally, *TTBK1* expression changes due to epigenetic modifications have been associated with AD progression [46].

The overexpression of *TTBK1* in ocular samples from POAG patients indicates a potential involvement of this gene in neurodegenerative pathways that are common to both glaucoma and AD.

While several studies have explored an omics approach in glaucoma, particularly transcriptomic analysis, this study offers a novel contribution by establishing an integrative link between visual, cognitive, and molecular profiles. This multidimensional perspective provides fresh insights into the systemic nature of the disease and lends biological plausibility to the observed clinical associations between glaucoma and cognitive decline.

Overall, the convergence of structural retinal changes, cognitive impairment, and transcriptional signatures of neurodegeneration supports the reclassifying of glaucoma as a neurodegenerative disorder rather than merely a condition confined to the eye. While this study establishes key associations, the causal relationships require further elucidation. Future research, employing longitudinal designs and neuroimaging techniques, may clarify the temporal dynamics of cognitive decline in glaucoma. In addition, functional studies of TTBK1 and CTGF/CCN2 in ocular surface samples and in retinal and neural tissues could help validate their roles mechanistically.

Although the conjunctival epithelium is not the primary site of glaucomatous damage, the changes in gene expression observed may reflect broader ocular or systemic alterations associated with POAG. In particular, this study revealed an increased expression of neurodegenerative genes that are normally not elevated in healthy conditions, suggesting ongoing tissue remodeling that parallels similar processes in the trabecular meshwork and optic nerve head. Therefore, several studies have explored conjunctival and tear film, non-invasive indicators on the ocular surface in ocular pathologies such as diabetic retinopathy, age-related macular degeneration, and glaucoma [47,48,49,50].

Our findings contribute to a growing body of evidence that positions glaucoma as a systemic neurodegenerative condition with both ocular and cerebral implications. The alignment of visual, cognitive, and molecular data emphasizes the need for an interdisciplinary approach in the diagnosis and management of glaucoma. This could lead to developing neuroprotective strategies that benefit not only the eye but also the brain [51]. Notably, several omics studies have reported CTGF, senescence pathway, the AD pathway, and neurotrophic factors [37,52,53,54].

Consequently, the identification of neurologically relevant biomarkers, such as *TTBK1* and *CCN2*/*CTGF* from ocular samples of POAG patients, supports their potential inclusion in future diagnostics. The alignment of visual, cognitive, and molecular findings underscores the importance of an interdisciplinary approach to diagnosing and managing glaucoma, which could lead to novel neuroprotective strategies addressing both ocular and cerebral degeneration, improving outcomes for patients with glaucoma. Moreover, our study revealed cognitive decline in POAG patients, despite no significant age differences between groups. This finding suggests that cognitive impairment in POAG may be intrinsically linked to the disease process rather than to age-related decline. Accordingly, early screening for cognitive dysfunction in glaucoma patients may be warranted as part of comprehensive care.

Despite these insights, a significant limitation of this study is its relatively small sample size, which may undermine the generalizability of the findings and the statistical power to detect more subtle associations, especially in the molecular analysis. While the integrative approach that combines visual, cognitive, and molecular data provides valuable insights, larger and more diverse cohorts are essential to validate these results and address interindividual variability in disease presentation and progression. Furthermore, although we included POAG patients, we did not classify them according to the severity of glaucoma progression. We recommend that future researchers classify patients to identify molecular and cognitive function links that may indicate potential biomarkers for glaucoma, based on severity.

Importantly, in this study, which includes POAG patients and controls without significant age differences, we found that POAG patients exhibited lower cognitive levels. Likewise, the structural and functional visual parameters attributed to the disease are not influenced by age. Furthermore, only POAG patients with no prior history of CNS diseases were included; thus, we associated these genes, RNA-seq analysis, and cognitive impairments directly with glaucoma, as noted in previous research. We also recommend that future studies stratify participants by glaucoma severity and the control group for possible CNS comorbidities.

To the best of our knowledge, this is the first study conducted in Latin America that simultaneously integrates clinical, molecular, and cognitive approaches to glaucoma. This multidimensional perspective provides a broader understanding of the pathophysiological mechanisms involved in the disease, as well as their functional implications in patients’ daily lives. Future multicenter studies in Latin America are warranted to validate and expand upon these findings.

Future studies should consider expanding the panel of molecular biomarkers beyond *TTBK1* and *CTGF/CCN2* to include additional candidates implicated in neurodegenerative pathways. Incorporating proteomic and metabolomic data may enhance understanding of glaucoma as a systemic neurodegenerative condition and help identify early diagnostic indicators or therapeutic targets.

## 4. Materials and Methods

### 4.1. Study Subjects and Clinical Data

The case–control analysis of the cross-sectional observational study involved patients with POAG and healthy individuals (Table 7). A non-probabilistic sampling method was used to select the sample. Ethics approval was granted by the Human Research Ethics at CES University (#261). The study adhered to the tenets of the Declaration of Helsinki and all participants were informed about the study objectives.

All individuals were recruited in Medellín City (Colombia). POAG individuals were diagnosed at the Clinica Oftalmológica de Antioquia (CLOFAN), using the following criteria: visual field (VF) defect, determined using a Humphrey Field Analyzer (Carl Zeiss Meditec Inc., Dublin, CA, USA) with the central 24–2 threshold test using SITA-standard strategy and elevated intraocular pressure (IOP) (>21 mmHg) in at least one eye. In addition, optical coherence tomography (OCT) (Cirrus HD-OCT, Zeiss, Dublin, CA, USA), involved measuring peripapillary RNFL thickness, optic nerve head (ONH) analysis, and macular Ganglion Cell Complex (GCC) assessment. In addition, other visual functions were performed, including visual acuity, color vision, ocular surface assessment, and ophthalmoscopy.

### 4.2. Neuropsychological Assessment

A comprehensive battery of standardized neuropsychological tests was administered to evaluate multiple cognitive domains, including attentional processes, visuospatial perception and construction, visual memory, and both semantic and phonological verbal fluency.

To assess visuospatial abilities and visual memory, the Rey–Osterrieth Complex Figure Test (ROCFT) was employed [55]. Attention, inhibitory control, mental flexibility, and psychomotor speed were evaluated using the Trail Making Test (TMT) [56]. Verbal fluency was assessed through both phonemic (e.g., “F”, “A”, and “S”) and semantic (e.g., “animals” or “fruits”) tasks [57]. Additional details regarding each test are described below.

#### 4.2.1. Rey–Osterrieth Complex Figure Test (ROCFT)

The ROCFT is a complex geometrical figure, and it is one of the most classical and widely used neuropsychological tests [58], requiring visuospatial construction and perception ability, visual (non-verbal) memory skills, attention and concentration levels, and fine-motor coordination [59]. The test involves two main conditions: (1) a copy trial, in which participants reproduce a complex geometrical figure while viewing it, and (2) a delayed recall trial, typically administered after 3 or 30 min, in which they draw the figure from memory. Scores for both trials range from 0 to 36. In this study, participants were provided with A4-sized paper and colored pencils (red, green, blue, and black) to track the sequence in which elements were drawn. During the copy condition, they were instructed to replicate the figure freehand. After a 3 min delay, they were asked to reproduce the figure from memory in the delayed recall condition.

#### 4.2.2. Trail Making Test (TMT)

Originally developed as part of the Army Individual Test Battery (1944), the TMT was later incorporated into the Halstead–Reitan Neuropsychological Battery (Reitan & Wolfson, 1985 [56]). The test consists of two parts:

- TMT-A evaluates processing speed, visual scanning, sustained attention, and motor control. Participants are instructed to connect 25 consecutively numbered circles distributed randomly across an A4 sheet as quickly as possible.
- TMT-B assesses divided attention, task-switching, working memory, and cognitive flexibility. Here, participants alternate between numbers and letters in ascending order (e.g., 1-A-2-B-3-C, etc.). Completion time (in seconds) for each part was recorded.

#### 4.2.3. Verbal Fluency Tasks

Verbal fluency was assessed in two formats:

- Phonemic Fluency: Participants were asked to produce as many words as possible beginning with the letters “F”, “A”, and “S” within a one-minute period for each letter, excluding proper nouns and morphological variants. This was assessed using the FAS Test [57].
- Semantic Fluency: Participants were instructed to generate as many items as possible within 60 s that belong to a specific semantic category (e.g., “animals” or “fruits”). The total number of valid words produced in each condition was recorded.

### 4.3. Sample Collection, RNA Extraction, Library Construction, and RNA-seq

Biological samples were collected from all participants under sterile conditions, in accordance with institutional ethical guidelines. Conjunctival epithelial cells were obtained from the superior bulbar conjunctiva via impression cytology, using sterile cellulose acetate filters, with the procedure conducted under topical anesthesia (OQ-SEINA- Oftalmoquimica). All samples were promptly transferred to the laboratory and either processed immediately or stored at −80 °C until RNA extraction.

Total RNA was extracted from conjunctival samples using TRIzol reagent (Invitrogen; Thermo Fisher Scientific, Inc., Waltham, MA, USA), following the protocol with slight modifications [60].

The concentration of total RNA was measured using Qubit 4.0 (Thermo Fisher Scientific, Waltham, MA, USA), and RNA integrity number (RIN) > 7 was determined with a High Sensitivity RNA assay, indicating suitability for downstream applications such as quantitative PCR or RNA sequencing. Additionally, RNA quality was analyzed through agarose gel electrophoresis, and RNA purity was confirmed with A260/280 ratios between 1.8 and 2.1.

Library fragment sizes were analyzed with the D1000 ScreenTape assay on the TapeStation System (Agilent Technologies, Santa Clara, CA, USA). We employed RNA sequencing technology to map gene expressions in ocular samples. For each sample, 200 ng of total RNA was used as input for the mRNA enrichment process, which included ribosomal RNA (rRNA) depletion to reduce background and increase the proportion of mRNA available for analysis.

Following rRNA depletion, library preparation was performed using an RNA Library Prep Kit, starting with enzymatic fragmentation to generate fragments of approximately 250 base pairs, followed by reverse transcription and cDNA synthesis. After, cDNA ends were repaired, sample indexing was conducted by ligating specific adapters and the libraries were subsequently amplified via PCR.

At each stage, magnetic bead-based purification was performed to ensure the specificity and quality of the products for the subsequent steps.

For the sequencing process, a circularization step was performed to convert the cDNA library into circular cDNA containing MGI adapters sequences. Circularization involved library denaturation, followed by ligation of the split oligo, enzymatic digestion, and purification using specific magnetic beads.

Subsequently, the process of DNA nanoball (DNB) generation was carried out. The circularized cDNA served as a template for the cyclic synthesis of its complementary strand, resulting in the formation of DNA nanoballs (DNBs) through Rolling Circle Amplification (RCA). These nanoballs range in size from 200 to 220 nm.

The resulting DNBs were then quantified and loaded onto a flow cell (FCL_PE100) containing a predefined pattern of spots for sequencing. The sequencing was conducted on a DNBSEQ-G400 platform, yielding a specific number of reads per sample.

Raw sequence reads were evaluated for quality using FASTQC; low-quality reads and adapter sequences were removed using Trimmomatic, retaining reads with Phred scores ≥30 and lengths ≥50 pb. All reads were mapped to the GRCh38 reference genome using STAR software (Version 2.7.11a), and aligned reads were stored in BAM format and sorted with SAMtools software (https://www.htslib.org/download/, accessed on 1 November 2024, Version 1.22.1). Counts for RefSeq genes were obtained using Feature Counts software (Version 2.7.15a) with the default settings.

### 4.4. Gene Expression Data Analysis

Differential gene expression analysis was conducted in R (v4.3.x), among various sample POAG group and control using DESeq2 packages from Bioconductor (v1.38.0) [61]. Genes with an adjusted *p*-value (Benjamin–Hochberg method) of <0.05 and an absolute log2 fold change ≥1 were considered significantly differentially expressed. To visualize the expression profiles, volcano plots and heatmaps were generated and Pearson correlation coefficient were calculated in R to assess the concordance in log2 fold changes between orthologous.

Functional annotation and clustering analysis of all DEGs were conducted using gene ontology (GO) and Kyoto Encyclopedia of Genes and Genomes (KEGG) analysis. The present study utilized the EnrichR software (https://maayanlab.cloud/Enrichr/, accessed on 10 December 2024) to conduct gene enrichment analysis [62]. Enrichment outcomes were determined based on a combined score exceeding 15, calculated by EnrichR using *p* value (Fisher’s exact test) and Z score (correction to the test). Subsequently, the enrichment results were presented as the percentage of genes enriched within each ontology category.

### 4.5. Real-Time Quantitative PCR (RT-qPCR)

The expression levels of DEGs identified through RNA-seq analysis were validated using quantitative reverse transcription PCR (RT-qPCR). A total of 200 ng of RNA from conjunctival epithelial cells was utilized for reverse transcription (RT) from seven samples (five POAG and two controls). SuperScript III First-Strand Synthesis SuperMix (qRT-PCR#11752-50, Invitrogen, Carlsbad, CA, USA) was employed, followed by quantitative polymerase chain reaction (qPCR) using Express SYBR GreenER (A10314, Invitrogen) on the Rotorgene 6000 Real Time Cycler™ (Corbett Research, Sydney, Australia), in which a 2-mL aliquot of each sample was added to the PCR mix. The qPCR conditions included an initial step of 10 min at 94 °C for predenaturation, followed by 35 cycles of 20 s at 95 °C, 15 s at 62 °C, and 30 s at 72 °C, and a final 10 s extension at 72 °C. The quality of the PCR products was assessed using 2% agarose gel electrophoresis (Thermo Fisher Scientific, Waltham, MA, USA).

For the relative quantification of gene expression using qPCR, both internal and external controls were employed. An internal control, specifically a housekeeping gene (18S ribosomal) was utilized to normalize the gene expression of interest. Additionally, external controls were included, and cDNA was obtained from two different control samples. All reactions were performed in duplicate, and the gene-specific primers for qPCR are listed in Table 8. The results were analyzed using the 2^−ΔΔ*CT*^ method [63].

### 4.6. Statistical Analysis

Data normality was assessed using the Shapiro–Wilk. Clinical data and qPCR results were recorded in Microsoft Excel and subsequently analyzed using SPSS software 27.0 (IBM Inc., Armonk, NY, USA). Comparisons between the study groups (POAG and control) were performed using either the T-Student’s or the non-parametric Mann–Whitney U test. All data are presented as mean values ± standard deviation (SD). A multiple linear regression analysis was conducted to evaluate the association between cognitive test results and glaucoma after adjusting for age (in years) and male gender as independent variables in the model. Pearson correlation coefficients (*r*) were calculated to assess clinical correlations between continuous variables, and their significance was determined using the corresponding *p*-value.

Additionally, GraphPad Prism software version 6.07 (GraphPad Software, Inc., Boston, MA, USA) was used to support the statistical analysis and generate figures. A *p*-value < 0.05 was considered statistically significant.

## 5. Conclusions

This study provides evidence that glaucoma is not merely an ocular disease but also displays key characteristics of a systemic neurodegenerative disorder. By integrating transcriptomic profiling with visual and cognitive assessments, we demonstrate a multidimensional relationship among retinal structural damage, cognitive impairment, and gene expression alterations associated with neurodegeneration.

The observed thinning of the RNFL correlates not only with visual field loss but also with deficits across various cognitive domains, particularly visual memory and executive functions. Furthermore, the identification of DEGs, such as TTBK1 and CTGF/CCN2, which are implicated in neurodegenerative pathways, supports the biological overlap between glaucoma and diseases like Alzheimer’s. These findings underscore the necessity of conducting larger-scale longitudinal studies to further investigate the genetic and metabolic pathways underlying cognitive impairment in POAG. Specifically, research utilizing ocular surface samples may offer a minimally invasive window into systemic neurodegenerative processes.

## Figures and Tables

**Table 1:** Sociodemographic and Ophthalmic Characteristics of Study Participants.

Variable	Control Group (*n* = 20) (Mean ± SD)	POAG Group (n = 20) (Mean ± SD)	*p*-Value
Age (years)	54.81 ± 6.05	59.76 ± 10.66	0.098
Sex (male/female)	17 (85%)/3 (15%)	10 (50%)/10 (50%)	
Level Education	*n* (%)	*n* (%)	**<0.001**
Low		3 (15%)	
Medium	8 (40%)	9 (45%)	
High	12 (60%)	8 (40%)	
IOP (mmHg)	17 mmHg ± 2.64	19 mmHg ± 6.81	0.231
Visual Acuity	0.48	0.10	0.021

SD = standard deviation and IOP = intraocular pressure. Bold formatting: statistically significant difference.

**Table 2:** Comparison of OCT structural and visual field functional parameters between control and glaucoma patients.

Parameter	Control Group (Mean ± SD)	POAG Group (Mean ± SD)	Statistical Significance (*p*-Value)
**OCT Structural Parameters**			
Average RGC	89.07 ± 9.31	84.10 ± 12.85	0.213
Superior RGC	88.77 ± 8.92	83.33 ± 13.87	0.168
Inferior RGC	87.85 ± 10.18	83.30 ± 10.25	0.298
Average RNFL Thickness (µm)	112.80 ± 11.17	95.46 ± 15.73	**<0.001**
RNFL Thickness Sup (µm)	117.00 ± 17.78	93.46 ± 21.99	**0.001**
ONH	0.45 ± 0.19	0.62 ± 0.16	**0.024**
Vertical C/D	0.49 ± 0.15	0.69 ± 0.15	**<0.001**
Rim Area (mm^2^)	1.35 ± 0.29	1.24 ± 0.37	0.321
Disc Area	2.00 ± 0.35	2.47 ± 0.45	**0.001**
Cup Volume	0.16 ± 0.35	0.45 ± 0.23	**<0.001**
**Visual Field Functional Parameters**			
Mean Deviation (MD) (dB)	−2.04 ± 1.92	−5.20 ± 6.06	**0.036**
Visual Field Index (VFI) (%)	98.01 ± 5.66	87.23 ± 12.35	**0.035 _a_**
Pattern Standard Deviation (PSD) (dB)	2.55 ± 1.11	3.80 ± 1.89	**0.019**

_a_ Chi-squared test. Bold formatting: statistically significant difference.

**Table 3:** Correlation between OCT structural measurements and visual field parameters in glaucoma patients.

OCT Parameter	VF Parameter	Correlation Coefficients (r)	*p*-Values
Average RNFL thickness (µm)	MD (dB)	0.36	0.190
Average RNFL Superior (µm)	MD (dB)	0.33	0.206
Average RNFL thickness (µm)	PSD (dB)	−0.53	0.032
Average RNFL Superior (µm)	PSD (dB)	−0.46	0.069

**Table 4:** Comparison of cognitive domain performance between control and glaucoma groups.

	Control Group	POAG Group	
	(*n* = 20)	(*n* = 20)	
	Mean ± SD	Mean ± SD	*p*-Values
ROCF copy	30.95 ± 3.31	27.06 ± 5.83	**0.015** ^β,^*
ROCF memory	18.75 ± 4.03	13.76 ± 4.90	**0.001** ^β,^*
TMT A (s)	51.40 ± 13.92	71.57 ± 21.06	**0.005** ^β,^*
TMT B (s)	103.60 ± 39.55	152.60 ± 42.69	**0.002** ^β,^*
TMT B-A	52.20 ± 34.09	76.08 ± 32.79	**0.048** ^α,^*
Fluency F	12.10 ± 4.36	8.90 ± 3.89	**0.019** ^β,^*
Fluency A	13.80 ± 4.11	10.50 ± 3.90	**0.013** ^β,^*
Fluency S	13.00 ± 4.06	10.50 ± 4.49	0.073 ^β^
Fluency Animals	20.55 ± 3.28	16.20 ± 4.48	**0.001** ^β,^*
Fluency fruits	14.40 ± 3.14	11.55 ± 2.72	**0.004** ^β,^*

ROCF: Rey–Osterrieth Complex Figure and TMT: Trail Making Test (A and B). ^α^ U Mann–Whitney. ^β^ T-Student. * *p* < 0.050. Bold formatting: statistically significant difference

**Table 5:** Correlations between neurocognitive performance and structural and functional visual measures.

Cognitive Domain	Neuropsychological Test	Associated Variable	r-Correlations	*p*-Values
Executive function				
Mental flexibility Inhibition response Perceptual-motor function	ROCF Copy	Fluency A	0.415	0.010
		MD (dB)	0.534	0.001
Mental flexibility Complex Attention (cognitive processing speed, inhibitory control)	TMT B	Fluency (animals)	−0.528	0.001
		Fluency (fruits)	−0.420	0.012
		Fluency F	−0.435	0.009
		Fluency S	−0.511	0.002
		RNFL Superior (µm)	−0.480	0.004
Learning and Memory (visuo-spatial memory)	ROCF Memory	Fluency (animals)	0.455	0.004
		Fluency (fruits)	0.472	0.002
		Fluency A	0.434	0.006
		TMT A	−0.441	0.009
		MD (dB)	0.529	0.001
		PSD (dB)	−0.591	0.000
Complex Attention (cognitive processing speed, inhibitory control)	TMT A	ROCF Memory	−0.441	0.009
		Fluency (animals)	−0.614	0.000
		Fluency (fruits)	−0.478	0.004
		Fluency F	−0.411	0.016
		Fluency A	−0.489	0.003
		PSD (dB)	0.409	0.016
Language (Fluency)				
	Fluency (animals)	RNFL (µm)	0.432	0.005
		RNFL Superior (µm)	0.428	0.006
		PSD (dB)	−0.411	0.008
	Fluency (fruits)	RGC thickness	0.460	0.003
		RNFL (µm)	0.462	0.003
		RNFL Superior (µm)	0.406	0.009
	Fluency A	RGC thickness	0.457	0.003
		RNFL (µm)	0.438	0.005
		RNFL Superior (µm)	0.405	0.010

r—Pearson correlation coefficient; *p*—*p*-value; indicates the statistical significance of the correlation, *p* < 0.05; TMT: Trail Making Test (A and B); ROCF: Rey–Osterrieth Complex Figure; MD: mean deviation (visual field); PSD: pattern standard deviation (visual field); RNFL: retinal nerve fiber layer; and GCC: Ganglion Cell Complex.

**Table 6:** Differential Gene Expression and functional roles of upregulated genes in glaucoma samples.

Expression Change	Symbol	Description	Gene Role	Log2 Fold Change
Upregulated Genes	*CCN2*/*CTGF*	Cellular Communications Network factor 2	It’s a downstream mediator of transforming growth factor beta (TGF-β) and modulates ECM homeostasis in the trabecular meshwork (TM). It’s upregulated in trabecular meshwork fibrosis and optic nerve head remodeling.	4.78
	*TTBK1*	Tau Tubulin Kinase 1	Overexpression is involved in autophagy and NF-κB signaling. TBK1 may cause retinal ganglion cell apoptosis and optic nerve damage contributing to neurodegeneration.	5.55

**Table 7:** Inclusion and exclusion criteria for the study participants.

Inclusion	Exclusion
Individual ages 39–70	Individuals under 39 years of age
Individual diagnosed with POAG, with or without drug treatment	Other glaucoma type
Individuals with best corrected visual acuity of 20/40	Individuals with neurodegenerative disease or disorders of the nervous system
In control group, non-glaucomatous healthy individuals, and not familiar history	Individuals with neurodegenerative disease or disorders of the nervous system, and hyperlipidemia or hypercholesterolemia.
	Individuals with glaucoma who present with macular degeneration, cataracts, or any other retinal pathology unrelated to glaucoma
	Individuals with retinal vascular occlusions of any etiology

POAG, primary open-angle glaucoma.

**Table 8:** Primers for the real-time quantitative PCR (RT-qPCR) assay.

Target	F/R	Primer Sequence (5′ → 3′)	Amplicon Size (BP)
TTBK1	Forward	TGGTGAGATCTACGAGGCCA	170
	Reverse	ACTTCTCGTTCCTGCCACAG	
CCN2	Forward	TTAGCGTGCTCACTGACCTG	182
	Reverse	GCCACAAGCTGTCCAGTCTA	

## Data Availability

The data presented in this study are available on request from the corresponding author. The data are not publicly available due to institutional privacy regulations.

## Abbreviations

The following abbreviations are used in this manuscript:

RGCs	Retinal Ganglion Cells
CNS	Central Nervous System
AD	Alzheimer Disease
IOP	Intraocular Pressure
POAG	Primary Open-Angle Glaucoma
SD	Standard Deviation
DEGs	Differentially Expressed Genes
RNFL	Retinal Nerve Fiber Layer
ONH	Optic Nerve Head
PSD	Pattern Standard Deviation
OCT	Optical Coherence Tomography
VFI	Visual Field Index
VF	Visual Field
TMT	Trail Making Test
sEVs	small Extracellular Vesicles
CCN2	Cellular Communications Network factor 2
TTBK1	Tau Tubulin Kinase 1
ECM	Extracellular Matrix
TGF-β	Transforming Growth Factor beta
TM	Trabecular Meshwork
GO	Gene Ontology
KEGG	Kyoto Encyclopedia of Genes and Genomes
RT-qPCR	Quantitative reverse transcription PCR
